# How are Treatment Decisions Made about Artificial Nutrition for Individuals at Risk of Lacking Capacity? A Systematic Literature Review

**DOI:** 10.1371/journal.pone.0061475

**Published:** 2013-04-16

**Authors:** Gemma Clarke, Katy Harrison, Anthony Holland, Isla Kuhn, Stephen Barclay

**Affiliations:** 1 CLAHRC End of Life Care, University of Cambridge, Cambridge, United Kingdom; 2 End of Life Care, City Care Centre, Cambridgeshire Community Services, Peterborough, United Kingdom; 3 Cambridge Intellectual and Developmental Disabilities Research Group, Department of Psychiatry, University of Cambridge, Cambridge, United Kingdom; 4 Cambridge University Medical Library, Addenbrooke’s Hospital, Cambridge, United Kingdom; 5 Department of Public Health and Primary Care, University of Cambridge, Cambridge, United Kingdom; Universidad Peruana Cayetano Heredia, Peru

## Abstract

**Background:**

Worldwide, the number of individuals lacking the mental capacity to participate in decisions about their own healthcare is increasing. Due to the ageing global population and advancing medical treatments, there are now many more people living longer with neurological disorders, such as dementia, acquired brain injuries, and intellectual disabilities. Many of these individuals have feeding difficulties and may require artificial nutrition. However, little is known about the decision-making process; the evidence base is uncertain and often ethically complex. Using the exemplar of artificial nutrition, the objective of this review is to examine how treatment decisions are made when patients are at risk of lacking capacity.

**Methods and Findings:**

We undertook a systematic review according to PRISMA guidelines to determine who was involved in decisions, and what factors were considered. We searched PubMed, AMED, CINAHL, EMBASE, PsychINFO, and OpenSigle for quantitative and qualitative studies (1990–2011). Citation, reference, hand searches and expert consultation were also undertaken. Data extraction and quality assessment were undertaken independently and in duplicate. We utilised Thomas and Harden’s ‘Thematic Synthesis’ for analysis. Sixty-six studies met inclusion criteria, comprising data from 40 countries and 34,649 patients, carers and clinicians. Six themes emerged: clinical indications were similar across countries but were insufficient alone for determining outcomes; quality of life was the main decision-making factor but its meaning varied; prolonging life was the second most cited factor; patient’s wishes were influential but not determinative; families had some influence but were infrequently involved in final recommendations; clinicians often felt conflicted about their roles.

**Conclusions:**

When individuals lack mental capacity, decisions must be made on their behalf. Dynamic interactive factors, such as protecting right to life, not unnecessarily prolonging suffering, and individual preferences, need to be addressed and balanced. These findings provide an outline to aid clinical practice and develop decision-making guidelines.

## Introduction

Decision-making capacity is a psychological construct which refers to a person’s ability to understand and balance the necessary information and to communicate a choice [1]. It has been enshrined in law in some countries, such as England and Wales (Mental Capacity Act, 2005). For adults, where consent is necessary for treatment to be lawful, capacity has become a pivotal issue in determining whether or not a person’s apparent wishes must be respected or, alternatively, the views of others are determinative. In practice an individual’s mental capacity may not be certain, or easy to establish. Capacity is decision-specific and varies over a person’s lifetime [2], [3]; incapacity in adult patients without severe mental illness can go unrecognised by clinicians [4]. Capacity may be affected by many factors such as intoxication from drugs or alcohol, traumatic brain injury, the presence of a developmental disability associated with an intellectual disability, or brain disorders such as dementia. Internationally, the ability of patients to participate in treatment decisions, such as whether or not to accept artificial nutrition, is becoming increasingly important. Many countries are now moving towards patient-centred models of shared decision-making [5]; for example, the United States and Canada are utilising multifaceted interventions to implement shared decision-making [6]. In England and Wales the Mental Capacity Act (2005) provides a framework for decision-making where someone lacks the capacity to make the decision for him/herself. It also enables the possibility for people to inform how, and by whom, decisions might be made on their behalf if they were to lose the capacity to make such decisions for themselves in the future. This includes the option of making a written advanced decision whilst having the capacity to refuse specified treatments in the future should capacity be lost. This is legally binding providing it is applicable, valid and relevant to the decision in question. This is particularly relevant to decisions about future artificial nutrition and hydration.

So what happens when patients lack the capacity to participate in treatment decisions, but have not previously made advance healthcare directives? The answers to such questions are set to increase in importance for clinicians internationally as the number of individuals at risk of lacking decision-making capacity is growing globally. The incidence of dementia is increasing as the age of the global population rises: the World Alzheimer Report estimates that the current 36 million people living with dementia will increase to 115 million by 2050 [7], [8]. Advances in medical treatment within high resource countries have also resulted in the increased survival of very low birth weight babies, a proportion of whom will have significant disabilities in childhood and later life [9], [10]. Improvements in healthcare have also resulted in increased life expectancy for those with intellectual disabilities [11], and those with acquired brain injuries such as stroke [12]–[14]. Decision-making for these individuals can present a number of practical, ethical and legal challenges [15]–[17]. It is particularly when questions of capacity arise with respect to the maintenance of life, or the end of life, as exemplified by decisions concerning artificial means for maintaining nutrition, that making healthcare choices may be the most difficult and contentious. This review uses the term artificial nutrition to refer to the administration of nutritious fluids through a tube for those unable to maintain adequate oral intake [18]. In the USA this may be referred to as ‘nutrition support therapy’ [19]. Many patients with neurological disorders, which put them at risk of lacking capacity, often have difficulties with oral feeding and may be considered for artificial nutrition. One UK study found that three-quarters of patients undergoing percutaneous endoscopic gastrostomy (PEG) insertion lacked the capacity to consent to this intervention [20]. Treatment decisions regarding artificial nutrition can be controversial due to; the uncertain prognosis of many undergoing interventions [21], the significant mortality and morbidity rates associated with interventions such as PEG [22], and a lack of evidence of benefit for PEG in certain patient groups, such as those with advanced dementia [23].

This review examines decisions about artificial nutrition made for three groups of individuals who demonstrate a range of issues surrounding decision-making capacity: those with dementia, those with intellectual disabilities, and those with acquired brain injuries. All three groups are vulnerable populations with potential long term feeding support needs. They differ in terms of: the permanence and reversibility of their capacity, feeding and mealtime requirements, and the ways in which they are perceived by wider society and within the healthcare system. The reason we are exploring these three groups is because the normal procedures of medical consent may not apply when a medical treatment takes place. Before a medical intervention is undertaken, it is standard good clinical practice for a healthcare professional to fully explain the procedure and its associated risks, and for the patient to give informed consent. For individuals with dementia, acquired brain injury or intellectual disability, who lack the capacity to make informed decisions, interventions may have to proceed without consent. It is the processes behind these decisions we are interested in exploring.

The overall aim of this systematic literature review was to review and synthesis the international evidence on treatment decisions concerning artificial nutrition for individuals at risk of lacking capacity due to dementia, intellectual disability, or acquired brain injury. With specific regard to:

- How decisions were made
- Who was involved in the decision-making
- Which factors were considered

These aims were achieved and are presented and discussed in the Results and Discussion sections below.

## Methods

Systematic reviews are increasingly incorporating qualitative and mixed methods data, [24], [25] as was the approach of this review. Reviews which incorporate evidence from both quantitative and qualitative research are particularly suitable for policy-related research as they maximise findings, provide a fuller picture and make the evidence relevant for policy-makers [26]. The review followed PRISMA (Preferred Reporting Items for Systematic Reviews and Meta-Analyses) guidelines (File S1) [27], which were adapted for a qualitative synthesis by drawing upon previously published qualitative syntheses on international public health and clinical practice issues [28]–[30]. This review was not registered.

### Search Strategy and Selection Criteria

Multiple search strategies were utilised. Six electronic databases were searched (PubMed, AMED, CINAHL, EMBASE, PsychINFO, OpenSigle) using criteria developed in collaboration with a librarian (IK). (Search strategy example in Materials S1). OpenSigle was selected as a grey literature database to search for unpublished studies and reduce publication bias. Citation and references searches were undertaken, as were hand searches of key journals; *BMJ, Lancet, PLOS Med, Gut,* and *J Pall Med*. The searches were undertaken from November to December 2011. The inclusion criteria were: research published between January 1990 and November 2011; any research design; original empirical data; any aspect of decision-making; any method of artificial nutrition; in which patients lacked, or were at risk of lacking, capacity. Language restrictions were not placed on search criteria. Non-English language papers were included if their abstract or a summary was available in English: if they passed abstract screening they were translated into English for consideration of inclusion in the review. Three such papers were included: two from The Netherlands and one from Japan. The exclusion criteria were: studies solely about preferences for artificial nutrition without regard to decisions; studies solely about artificial hydration; reviews, summaries and newspaper articles; and legal case studies. (See research protocol in Materials S2). Multiple papers from studies were included if they included additional original data.

The searches produced 9836 titles which were initially screened by one researcher (GC), 993 abstracts were independently reviewed by two researchers (GC, KH), and 165 papers were read in full by two researchers (GC, KH). Disagreements were resolved by discussion or in consultation with a third researcher (SB). The final number of included studies was 66. (Flow Chart illustrating the search process in File S2).

### Quality Evaluation

Gough’s Weight of Evidence framework [31] was employed to assess paper quality, relevance, bias and generate an overall judgement about contribution. Two researchers (GC, KH) independently weighted the studies; disagreements were resolved by discussion or with a third researcher (SB). Studies with all weights were included provided they met the minimal requirements for relevance and quality [32]. Studies rated ‘high’ were given greater weight; a sensitivity analysis examined the effect of removing lower quality studies.

### Analysis

In their influential work “Principles of Biomedical Ethics” Beauchamp and Childress [33] developed an approach to moral decision-making in medicine, based upon four principles they reasoned as being common to utilitarian and deontological thinking and common morality (Table 1). They reason that decision-makers need to determine the relevance and balance of these principles, seeking a solution that gives each principle appropriate weight. This approach of “Principlism” provides a biomedical ethical framework for the synthesis of the literature reviewed in this paper.

**Table 1:** Four key bio-ethical principles (after Beauchamp and Childress).

**Autonomy**	Respect for an individual’s right to determine what is done to them.
**Beneficence**	A duty to do things that will help others.
**Non-maleficence**	A duty to not do things that will harm others.
**Justice**	Respect for an individual’s right to equitable treatment with others.

A ‘thematic synthesis’ was used for data analysis and synthesis [26] as previously used in mixed-method systematic reviews [34]. Two researchers (GC, KH) extracted data from included papers into ‘descriptive themes’, which were then entered into NVivo 8 for further analysis and interpretation to yield ‘analytical’ themes by one researcher (GC).

## Results

The 66 included papers comprised data from 40 countries and 34,649 individuals including patients, carers, family members and clinicians (see Table S1). The majority of papers (49) involved people with dementia, 30 involved people with acquired brain injuries (ABI), and only four examined people with intellectual disabilities. Methods of artificial nutrition included: percutaneous endoscopic gastrostomy (PEG) (39), non-specific methods such as “tube feeding” (26), nasogastric feeding (11), and other methods such as radiologically-inserted gastrostomy, percutaneous endoscopic jejunostomy and parenteral nutrition (6). Most papers comprised data from North America and/or Europe, the largest number being USA (28) and the Netherlands (11). The sensitivity analysis revealed that removing studies rated as ‘low’ did not alter the findings. Six main themes emerged.

### Triggers for the Decision-making Process

Clinical indications alone were often insufficient for determining the outcomes of the ethically complex decisions surrounding tube feeding. However, changes in a patient’s condition were often the primary triggers for beginning the decision-making process [35]–[49]. For all three groups, weight loss [38]–[40], [47], observed changes in nutritional status [35], [38], [42], [44], [45], [50], [51], swallowing difficulties [38], [42], [45], [47], [48], other difficulties with feeding [40], [47], and food refusal [52]–[55], were sources of concern and prompted initial discussions.

For some individuals, an acute incident, such as a stroke or infection, brought them into emergency care where feeding difficulties were observed [42], [50], [56]–[61]. For others, changes occurred as a slow deterioration, particularly for individuals with dementia. Alongside dieticians [62]–[64] and speech and language therapists [44], nurses and nursing home staff played a pivotal role in initiating discussion about artificial nutrition. By noticing small changes during daily care, such as weight loss, nursing staff decide when to call for further help or alert the patient’s family [52], [65]–[68]. Nurses’ decisions to report observations were often based on a “*gut feeling*” that “*something was wrong*” [52]. Similarly for individuals with intellectual disabilities, mothers [37], [69] and care workers [39] brought changes to the attention of specialists. Conversely, for decisions concerning withdrawal, it was the lack of change in a patient’s condition that triggered the decision process [70]–[74].

### Quality of Life

Improving a patient’s quality of life was cited as the principal aim behind most decisions in the majority of studies for all patient groups [45], [46], [55], [66], [70], [72], [74], [75]. However, the concept of quality of life was interpreted differently across different cultures and contexts. It was primarily interpreted in one of two ways: ‘freedom from’ or ‘freedom to’.

In the first interpretation, quality of life was related to freedom from pain and discomfort. Within some studies, administering artificial nutrition was portrayed as interfering with the dying process [45], [46], [52], [66]. Thus forgoing artificial nutrition resulted in freedom from discomfort [54] and suffering [48], [67]. This perspective was particularly evident in the Netherlands [45], [52], [66] and Belgium [35], [36]. Similarly, for individuals with intellectual disabilities, some parents believed that a PEG tube would increase physical suffering and social stigma [39], [69]. Conversely, a smaller number of US and Canadian studies indicated that some clinicians believed starting artificial nutrition at the end of life could provide freedom from pain and discomfort, as feeding tubes were perceived to increase the ease of feeding and administering medicines [40], [76].

In the second interpretation, the concept of quality of life was portrayed as the freedom to enjoy life’s pleasures such as food despite any risks which may follow, for example choking on food. Many studies across North America and Europe cited the pleasure of eating as the main reason behind forgoing tube feeding in palliative contexts [47], [68], [77] and for younger people with intellectual disabilities [37], [69]. For parents of children with intellectual disabilities, oral feeding had a special significance; it was seen as “playful” “together time” for parent and child [37]. Despite the risk of aspiration, oral feeding was also cited as a social activity for both people requiring palliative care [77] and people with intellectual disabilities [37], [69]. One participant said:

*I think you come to a point when you accept risk and you save that person’s quality of life* [68] *.*

A further more basic interpretation was related to a patient’s level of consciousness. Patients in a persistent vegetative state were widely perceived as having a poor quality of life due to their minimal level of consciousness, which was frequently cited as the reason for withholding or withdrawing artificial nutrition [70].

### Prolonging Life

The second most cited, and contrasting decision-making factor, was prolonging life. This aim was particularly well established for elderly patients in Japan, but a similar situation was also found in Hong Kong [48], Israel [76] and within some US and Canadian studies [67], [78]–[81]. The literature revealed that in Japan, artificial nutrition at the end of life is common [49], [56], [58], [82], many physicians felt they must start artificial nutrition for the sake of the patient’s family [49], [56]. One physician stated:

*In a sense, Japanese people have to live not to fulfil their own happiness but their families’ when they fall into this situation* [56] *.*

In Japan, artificial nutrition was perceived as part of basic care; withholding it would be neglectful other than for medical reasons such as diarrhoea [56]. One study found artificial nutrition was prevalent across all elderly patients, but was significantly more likely to be started if the patient had dementia [83]. Two studies illustrated how the clinical environment in Japan promotes the use of artificial nutrition through the healthcare insurance system and fear of law suits [49], [56].

The clinical environment of some American nursing homes also promoted tube feeding for the primary purpose of prolonging life [65]. In one nursing home there was a belief that “African American families preferred aggressive end-of-life care, including tube feeding” [65]. Another US study of community members reported that both African-American and Caucasian individuals thought tube-feeding could help prevent a person “*starving to death*” [84].

### The Patient’s Wishes

Another key decision-making factor was the patient’s own wishes. The literature suggests that very few patients worldwide have advanced healthcare directives (AHD) regarding artificial nutrition. Of the studies which inquired about advanced directives, almost none [42], [46], [74], [85], or very few patients had made them [41], [45], [52], [55], [59], [66], [78], [80], [86]. However, AHDs were perceived as influential, by clinicians and family members [70], [71], [81], [86]–[88]. Only a minority thought family wishes should override a patient’s advanced healthcare directives [54], [88]. One physician said:

*…if the family says yes but we are enjoying life with dad so much and I see that he’s enjoying it too, then in that case I tend to be more active than the advance directive would seem to indicate. Yes. And I think that is totally justified* [86] *.*

Informal spoken wishes of patients were also thought to be important in the decision-making process [42], [66], [67], [84], [85], [89], [90]. However, verbal wishes expressed once a person’s disease had progressed were sometimes considered untrustworthy [54].Within studies of dementia and ABI, non-verbal signals, such as pushing food away, were frequently incorporated into an assessment of presumed wishes [35], [36], [47], [48], [52], [66], [68], [87]. Some even felt this was an expression of patient’s right to autonomy and should be decisively respected [67]. A smaller number of participants viewed such behaviour as a symptom of disease and advocated forced feeding or artificial feeding [38], [47], [54], [68]. In contrast, within studies of children with intellectual disabilities, non-verbal signals were not weighted with the same importance in the decision-making process [37], [39], [69].

### Family Role

Across all three patient groups, clinicians held the most power within the decision-making process. However, family members were influential and often acted as surrogate decision-makers for informed consent [44], [65], [67], [74], [86], [91], despite the stress and anxiety this caused [43], [46], [48]. Decisions to forego tube feeding at the end of life were generally associated with greater family input into the decision-making process, particularly in Belgium [35], [36] and the Netherlands [52], [66]. Conversely, studies which found starting artificial nutrition at the end of life was commonplace, also found a paternalistic medical culture in which families had less direct input into decisions. This was the primary model of decision-making found in Japan [49], [56], [58], [82], in one location within the USA [65], and partly in Hong Kong [48].

When family members were able to influence decisions, the style used was either: ‘best interests’, often only taking into account the patient’s physical condition [41], [47], [78], [80], [92], or ‘substituted judgment’ in which the surrogate made decisions based upon the patient’s presumed wishes [66], [84], [88], [92]. A smaller number of surrogates made reference to ‘what they would want for themselves’ [84], [88].

### Clinicians’ Conflicted Views

The majority of medical practitioners felt conflicted about their role. For example, one study of physicians in Japan found that despite the strong cultural incentives to provide artificial nutrition, 53% said they would refuse PEG for themselves [49]. Similarly, a survey of Israeli physicians revealed that while they would recommend PEG for dementia patients, 77% would not recommend it for their own relative in a similar situation [76].

Nurses found their role in decisions particularly stressful, often feeling conflicted because they considered the provision of food and water as to be part of their role, yet they were often limited in their ability to influence final decisions [35], [36], [40], [52], [67], [93]. Consequently, nurses might sometimes try to subtly or covertly influence decisions. One nurse stated:

*It happened [in a way in which] the physician probably did not wish. We contacted the family… actually a little bit behind the physician’s back... Then the family said: ‘we will take her home with us* [without ANH] [36] *.*

### Summary

Dementia: The majority of the studies (47) involved people with dementia. The decision-making process was triggered when an acute incident, or a slow deterioration in condition drew medical attention. The main motivation behind decisions, either to start or to forego, was to improve the person’s quality of life. In Asia and certain parts of the US, another key motivation was to prolong life. In most cases the patient’s informal spoken wishes and an interpretation of their behaviour were usually taken into account; less frequently behaviour and spoken wishes were thought to be a symptom of the person’s diseases and therefore disregarded. Family members were more active in decision-making in Europe and some parts of North America, than in Asia and certain parts of the USA. Active family participation was often correlated with the decision to forgo artificial nutrition.

Acquired brain injury: In the 28 studies identified, the key decision triggers and factors were similar to those for people with dementia, with the exception of patients in a persistent vegetative state (PVS). PVS patients were thought to have the lowest quality of life based upon their reduced level of consciousness and decisions to withdraw artificial nutrition were sometimes made, triggered by a lack of change in their condition.

Intellectual Disability: Only four studies were identified, revealing a lack of research in this patient group. Some additional decision-factors were identified: alongside quality of life and prolonging life, the social stigma of artificial nutrition and the special significance of oral feeding were important. Family members reported pressure from clinical staff and feeling guilty about starting artificial nutrition.

### Who was Involved in the Decision-making?

Fifty-six of the studies contained some data about who was involved in the decision-making. This varied greatly from doctors and relatives, to speech and language therapists, dieticians and multidisciplinary teams. Of studies which collected data on who had the most influential role in the decision-making process, 14 studies found one or more of the physicians to be the most influential [35], [36], [42], [44], [49], [52], [57], [58], [61], [65], [71], [74], [84], [94], nine found collaborative decision-making between the relatives or substitute decision-makers and members of the medical team [46], [50], [51], [65], [73], [86], [90], [95], [96], three studies found that the relative or surrogate decision-maker were the most influential or felt they had the final say [41], [59], [97], and one study found that the medical director was the most influential [98]. However, the real-life picture may be more complex than these findings indicate, as one study reveals that although surrogate decision-makers authorised the decision in 92.2% of the cases, a detailed discussion with the patient or surrogate was only documented in one of the 154 cases [59]. Although, doctors most frequently had the final say, family members and care-givers could influential in the process. For example, in one study doctors expressed how they took into account the families’ emotional responses when decision-making [66]. When families were involved in the decision-making process they felt more satisfied with the decision [96], and when they felt they weren’t involved at all with the process they felt unhappy, anxious and stressed [39], [61], [69]. For example, in one study a relative felt unhappy because:

*…the ward sister said to me, ‘You’ve got no choice now, it’s compulsory’, so I thought I’ve got no say in it* (21).

Decisions to forego tube feeding at the end of life were generally associated with greater family input into the decision-making process, particularly in Belgium [35], [36] and the Netherlands [52], [66]. Conversely, studies which found starting artificial nutrition at the end of life was commonplace, also found a paternalistic medical culture in which families had less direct input into decisions. This was the primary model of decision-making found in Japan [49], [56], [58], [82], in one location within the USA [65], and partly in Hong Kong [48].

Nurses were also indirectly involved in the decision-making process. They often acted as go-betweens for the decisions between the family and doctors [36]. Nurses found their role in decisions particularly stressful, often feeling conflicted because they considered the provision of food and water as to be part of their role, yet they were often limited in their ability to influence final decisions [35], [36], [40], [52], [67], [93]. Consequently, nurses might sometimes try to subtly or covertly influence decisions.

## Discussion

Our review of the international research evidence indicates that clinical indications for artificial nutrition are very similar across conditions internationally. However, observed changes to a patient’s conditions only trigger treatment decisions, and the decision-making process for those lacking capacity was often long and challenging. The main decision factor cited by the majority of participants internationally was improving the patient’s quality of life. However, the meaning of the term ‘quality of life’ was complex and variable, often divided between ‘freedom from’ and ‘freedom to’. This division can perhaps be seen as analogous to philosopher Isaiah Berlin’s classic concepts of negative and positivity liberty [99]. Berlin describes negative liberty as the absence of constraint, while positive liberty is the freedom to choose. Berlin highlights the need for balancing both the positive and negative aspects of liberty instead of conflating them, which may be a useful approach for thinking about quality of life within decisions regarding artificial nutrition.

The interaction among the themes is very complex and highly dependent on both the clinical condition and the socio-cultural context. In such a wide range of decision-making situations, it is therefore not possible to generate a unified over-arching framework by which to assess interventions or outcomes as good and adequate, beyond emphasising the need to ensure that the four bio-ethical principles are each given appropriate weight in each decision.

The second most cited factor was the explicit aim to prolong a patient’s life. This was particularly well established for elderly patients in Asian countries, but was also found within some studies in the USA and Canada. However, the situation in Japan is changing: in January 2012 the Japan Geriatrics Society changed its position statement for the first time in 11 years. It now aims to promote the withholding or withdrawal of artificial nutrition as a valid healthcare choice for elderly patients [100]. This change is part of a range of reforms of elderly care in Japan, the objective of which is to reduce the number of people on artificial nutrition, particularly those with advanced dementia. This will bring Japanese policy closer to the policies currently in place in Europe and the USA. While this review revealed considerable cultural and international differences, it is acknowledged that one limitation of this review is that fewer studies were located from Asia (7), the Middle East (1). No studies from Africa were located. This may have limited the ability for the international differences to be elucidated further.

The majority of studies contained evidence about who was involved in the decision-making, and what factors they considered. However, only four studies examined decision-making in practice [48], [52], [65], [66]. This means that although some data was found, our research aim (a) to examine ‘how decisions were made’ was the least fulfilled of the objectives. Future qualitative research could build upon this finding by observing clinicians and relatives discussing and negotiating decisions. Three studies examined gender and its relationship to decision-making within the family. A study of Chinese-American caregivers for relatives with dementia found that female decision-makers often felt bound by the traditional gender role of “wifely deference” [101]. Two studies examining parental decision-making for children with intellectual disabilities found that women experience feelings of guilt and failure as mothers [37], [69]. Although many studies presented demographic data about the gender of participants, no other studies used gender as a unit of analysis or explored it qualitatively. The theme of gender could perhaps be developed further in future research studies. There were just four studies pertaining to decision-making about those with intellectual disabilities located, further research into this area is required.

Only eight studies described or discussed the process of assessing the patient’s decision-making capacity [38], [42], [43], [43], [47], [53], [59,97]. Within those studies that did utilise a capacity assessment, the Mini-Mental State Examination was the most frequently used [38], [43], [47], [53]: whilst this is an established measure of the severity of dementia it does not directly assess a person’s ability to make a particular decision. This lack of consideration of capacity as a concept across the literature, or an understanding of how it is assessed, was surprising as the review question was focused on capacity issues. This may reflect the fact that studies focusing exclusively on assessing a patient’s mental capacity were excluded if they did not contain evidence about how decisions were made, who was involved or what factors were considered. Alternatively, this may reflect the fact that thinking about capacity and legislation incorporating this concept are relatively new: in England, for example, the Mental Capacity Act 2005 only came into force in 2007. While decision-making capacity does not influence *when* the decision to start clinical intervention should be made, or *whether* it should be made, it does fundamentally influence *how* that decision is made, and crucially how much the patient themselves can participate in the decision making process. However, as the global population at risk of lacking the capacity to participate in treatment decisions rises, a greater focus on these issues within clinical practice will be needed. Future studies on decision-making could build upon these findings by including mental capacity testing as part of their research methodology or by asking clinicians about how they assess capacity for those at risk.

### Implications for Clinical Practice

The recommendations which arise from this review are fundamental to good clinical practice; however, the literature indicates that these necessary elements may not be happening in all cases. The recommendations are based around a conceptual framework of; the indicators which alert clinicians to the need to make a decision, who is then subsequently, involved in the decision-making, and what factors are considered to make the decision. These are outlined below.

- At the beginning of the decision-making process, discussions should be held with nurses and staff who care for patients on a daily basis. As the evidence shows these individuals are central in making early observations about small changes in a patient’s condition or behaviour regarding eating, drinking, weight loss and behaviour.
- Feeding difficulties may not be identified in an acute context. Regular checks of at risk patients should be undertaken.
- Clinical indications start the decision-making process for artificial nutrition but are rarely adequate to make the final decision. Emotional, social and ethical factors should always be considered when making decisions about artificial nutrition. Guidelines appropriate for the local legal and social customs should be drawn up to assist clinicians in discussing and balancing these sometimes conflicting factors.
- The most important non-clinical decision-making factor worldwide was quality of life. This should be of central consideration to all decisions made. It may involve balancing potential risks, such as choking, with benefits, such as enjoying mealtimes.
- Regular meeting with family and next of kin should be held to improve communication and decision-making processes.
- Emotional, social and ethical factors, such as ‘quality of life’, can vary in meaning. All those involved in the decision-making process should be encouraged to speak freely and be explicit about their aims to ensures that all those involved in the process have a shared understanding before proceeding in each stage of the process.
- Few patients worldwide have advanced directives regarding artificial nutrition: their important influence in decision-making means that where possible, individuals should be encouraged to create advanced directives about their future care.
- Clinicians, and in particular nurses, found their involvement in decisions stressful. Greater support may be required for healthcare workers involved with decisions; clear guidance and policies may reduce conflicted feelings
- The legal requirement for decision-making capacity testing and the informed consent standard will vary depending upon the jurisdiction. However, as an increasing number of patients are at risk of lacking capacity, a greater focus on mental capacity within clinical practice will be required, with education for clinicians in all specialities.

When people have full decision-making capacity concerning artificial nutrition, which is commonly the case during treatment for head and neck cancer or in Motor Neurone Disease, it is for the clinical team to put the options forward and for the patient to decide. Personal factors and cultural and religious beliefs may influence whether or not they choose to opt for artificial nutrition. However, when it comes to people who never had the capacity to fully consider these issues, or who had the capacity at one time but have now lost it through illness, decisions are more complex. The challenge is how to protect their right to life, but at the same time not prolong it in a manner that causes further suffering; and how to know and reflect individual wishes within the framework of the law of their country.

## Supporting Information

Table S1 Cross-tabulation illustrating study populations, methods, key findings and weight of evidence
(DOCX)

File S1 PRISMA Checklist
(PDF)

File S2 Flowchart illustrating the search process
(DOCX)

Materials S1 Example of one search strategy
(DOCX)

Materials S2 Research protocol
(DOCX)
