# Supplementary material for: How are Treatment Decisions Made about Artificial Nutrition for Individuals at Risk of Lacking Capacity? A Systematic Literature Review
Source: PLoS One. 2013 Apr 16;8(4):e61475. doi: 10.1371/journal.pone.0061475 (PMC3628879; doi:10.1371/journal.pone.0061475)
Supplement: Table S1 — Cross-tabulation illustrating study populations, methods, key findings and weight of evidence (DOCX) [file pone.0061475.s001.docx]

**Table S1. Cross-tabulation illustrating study populations, methods, key findings and weight of evidence**

| **No.** | **First author and reference** | **Country** | **Setting** | **Research methods** | **Key findings** | **Weight of evidence** |
| --- | --- | --- | --- | --- | --- | --- |
| 1 | Aita, K. *BMC Geriatr;* 2007, 7: 22. | Japan | - Physicians (N.30) - Starting and withholding decisions - PEG feeding - Dementia patients | Semi-structured qualitative interview | - 26 physicians found it difficult to offer option to withhold, only 4 offered this to families - Factors which favour initiating PEG: clinical environment, legal consequences, emotional reluctance cultural values, financial system - Factors which allow option to withhold: communication with family, build relations with family, team decisions, offer alternatives | H H H - **H** |
| 2 | Aita, K. *Arch. Geron. & Geriatr*, 2008; 46(3):263-272. | Japan | - Physicians (N.27) - Starting, withholding and withdrawing decisions - ANH non-specific - Dementia and ABI patients | Semi-structured qualitative interview | - ANH is compared to food and water, withholding it is considered neglectful - ANH is cheaper - ANH is low technology and look ‘less’ artificial so is less likely to be withheld - Physicians feared legal consequences and negative media coverage if withholding | H H M - **H** |
| 3 | Bell, C. *J Palliat Med* 2008; 11(6):915-924. | USA | - Physicians (N.388) - Starting, withholding and withdrawing decisions - ANH non-specific - Dementia and ABI patients | Postal structured questionnaire survey, using a patient vignette | - Most physicians (69%) chose to withhold for post-stroke patient with uncertain prognosis - A living will was more important to physicians who chose to withhold (95%) than those who started (80%) - Patient’s verbal preferences important to both those physicians who would withhold (97%) and start (88%) - Family preferences more important to those started (55%) than withheld (20%) - Liability rated important by those who would start (52 %) than those who would withhold (28 %) | H H M - **H** |
| 4 | Bryon, E*. Int J Nurs Stud*, 2010; 47(9): 1105-1116. | Belgium | - Nurses (N.21) - Starting, withholding and withdrawing decisions - ANH non-specific - Dementia patients | Semi-structured qualitative interview | - Nurses are intensively involved in the care surrounding ANH decisions - Nurse build up detailed picture of patient and play a communicative and preparatory role in decisions. - Nurses may disagree with some decisions made for patients, they may resist with either open disagreement or hidden tactics | H H H - **H** |
| 5 | Bryon, E. *Bioethics*, 2011; (epub) 1467-8519. | Belgium | - Nurses (N. 74 questionnaires, N.21 semi structured interview) - Starting, withholding and withdrawing decisions - ANH non-specific - Dementia patients | Semi-structured qualitative interview and written questionnaires | - Nurses affected by persistent signals from patients such as refusal of food - Personal involvement with the patients created empathy and with the patients - Nurses felt their involvement with decisions was ‘obvious’ due to their intimate nurse-patient relationship - Nurses participation in decisions depended upon their individual relationship with the physician | M H H - **H** |
| 6 | Buiting, H.M.*Palliative Medicine,* 2011; 25(1): 83-91. | Netherlands and Australia | - Physicians (N.31) - Starting, withholding and withdrawing decisions - ANH non-specific - Dementia patients | Semi-structured qualitative interview | - Both Dutch and Australian doctors tried to interpret whether ANH will improve quality of life and whether it is patient’s presumed wish - Both D & A doctors thought it important for all involved in the decision to be happy (incl. family, patient) - All participants felt ANH interferes with dying process - Food refusal was seen as indicative of patient’s wishes – particularly by Dutch doctors - Australian doctors felt advance directives were more important, Dutch doctors felt discussion with family and advance care planning was most important | H H H - **H** |
| 7 | Ely, J.W.*J Am Geriatr Soc*, 1992; 40(5): 471-475. | USA | - Family doctor (N.439) - Starting, withholding decisions - PEG feeding - ABI patients | Postal questionnaire using patient vignette | - The family’s opinion was the most important factor - Most physicians (59%) said a living will was important but it only had limited influence in the vignette - Physicians who received the standard living will were less likely to change their minds than those who received a specific one (20% vs. 40%) - 50% of those who initially wanted PEG would change their minds if the patient’s condition did not change within a year | M H H - **H** |
| 8 | Golan, I. *Israel Med Assoc Jour* , 2007; 9(12): 839-842 | Israel | - Physicians (N.72), family of patients (N.126), gastroenterologists (N.34) - Starting decisions - PEG feeding - Dementia patients | Closed question questionnaire | - For most physicians (85%) improving quality of life was the most important factor, followed by preventing aspiration (82%) - Only 23% of physicians would recommend the procedure to a relative in the same situation - 63% of guardian felt they were part of the decision-making process | H H H - **H** |
| 9 | Grubb, A. *The Lancet*, 1996; 348(9019): 35-40. | UK | - Physicians (N. 1027) - Withholding, withdrawing decisions - ANH non-specific - ABI patients | Postal questionnaire | - 94% of physicians would withdraw if the patient were to remain vegetative - 39% of physicians thought advance directives should be decisive, 46% thought contributory - 12% thought patient’s previous informal opinion should be decisive, 58% thought contributory - 21% thought either spouse or parent opinion was decisive., 67% thought contributory | H H M - **H** |
| 10 | Hodges, M.O. *Arch Intern Med,* 1994; 154(9): 1013-1020. | USA | - Medical interns (N. 326) - Starting, withholding and withdrawing decisions - ANH non-specific - Dementia and ABI patients | Postal questionnaire | - The main reason for opposing tube feeding in either dementia or PVS was the patient’s irreversible disease (68% dementia, 84% PVS) - The main reason for starting tube feeding in either dementia or PVS was the belief that it was a physicians role to sustain life (44% dementia, 42% PVS) | M H H - **H** |
| 11 | Lavrijsen, J. *Brain Injury*, 2005; 19(1): 91-102. | Netherlands | - PVS patients (N.5) - Starting, withholding and withdrawing decisions - PEG and NG feeding - ABI patients | Qualitative in-depth description case studies | - In all 5 cases, length of time in vegetative state and futility of treatment were key decision factors - In 3 cases problems with NG prompted PEG discussion - Ultimate decisions remained up to physician though family wishes were always taken into account - Decision-making was a learning process within each nursing home | M H H - **H** |
| 12 | Lopez, R.P. *J of Clin Nurs,* 2010; 19(5-6): 632-639. | USA | - Nurses (N. 11) - General decision-making - ANH non-specific - Dementia patients | Semi-structured qualitative interview | - Nurses prompt decisions by reporting their observations about medical factors such as nutritional status, skin breakdown etc. - Nurses role in decisions is to facilitate communication, report observations and support the family - Nurse felt uncomfortable or avoided direct participation in decisions, sometimes opting for implicit communication | M H H - **H** |
| 13 | Lopez, R.P. *Arch Intern Med*, 2010; 170(1): 83-88. | USA | - Nursing homes (N. 2) - Starting decisions - PEG feeding - Dementia patients | Ethnography | - Nursing homes (NH) with low PEG usage had mission statements emphasising care for individuals - NH with high PEG usage had mission statements which emphasised high quality care - NH with low PEG usage emphasised communication with family - NH with high PEG usage, assumption families did not want to be involved with care decisions | H H H - **H** |
| 14 | Mitchell, S.L. *Can Med Assoc Jour,* 1999; 160(12): 1705-1709. | Canada | - Surrogate decision-makers (N. 46) - Starting, withholding decisions - PEG and PEJ feeding - Dementia and ABI patients | Patient chart review and telephone interview with both closed and open questions | - The most common reasons for starting AN were to prolong life (80.4%), to fulfil a moral obligation to the patient (78.3%) and prevent aspiration (71.7%) - In the majority of cases (39.1%) the physician made the decision, in 28.3% of cases it was joint decision-making, and in 32.6% of cases it was the surrogate alone | H H H - **H** |
| 15 | Mitchell, S.L. *J Am Geriatr Soc*, 2000; 48(4): 391-398. | Canada and USA | - Surrogate decision-makers (N. 94) - Starting, withholding decisions - PEG and PEJ feeding - Dementia, ABI and other patients | Patient chart review and telephone interview with both closed and open questions | - The most common reasons for starting AN were to prolong life (84%), to prevent aspiration (67%), and to fulfil a moral obligation (64.9%) - 39.4% of surrogates believed the tube feeding did improve the patient’s quality of life - Most surrogates (75.5%) in favour of AN at the time of the decision, 61.7% were in favour in retrospect - 54.3% surrogates felt supported in their decision, 70.2% did not feel pressured - 38.3%)would want tube feeding for themselves | M H H - **H** |
| 16 | Onwuteaka-Philipsen, B.D. *Age Ageing*, 2001; 30(6): 459-465. | Netherlands | - Nursing home physicians (questionnaire N.929, interview N.74) - Staring, withholding, withdrawing and continuing decisions - ANH non-specific - Dementia, ABI and ID patients | Postal questionnaire with closed questions and semi-structured qualitative interviews | - The discussion was initiated for 90% of all patients for medical reasons relating to the patients condition - Low quality of life (58%) and not unnecessarily prolonging life (58%) were the key factors in forgoing - Other important factors in forgoing were no prospect of improvement (505) and medical futility (38%) | H H H - **H** |
| 17 | Pang, M.C.S. *Jour of Nutr Health & Aging,* 2007; 11(6): 495-501. | Hong Kong and USA | - Dementia care hospital units (N.2) - Starting and withholding decisions - PEG, NG and PN feeding - Dementia patients | Ethnography | - Differences are not individual choice but a result of the predominant values embedded in the medical cultures - In the USA, respecting patient autonomy when forgoing was important. Advance directives were considered important - In the USA, place of care was considered more important than AN decisions - In Hong Kong, AN was considered basic sustenance | H H H - **H** |
| 18 | Pasman, H.R.W. *Alz Dis & Assoc Dis*, 2004; 18(3): 154-162. | Netherlands | - Nursing home physicians (NHP) (N. 178), nurses (N. 167), family members (N. 128) - Starting and withholding decisions - ANH non-specific - Dementia patients | Questionnaire containing closed questions | - The key factor in forgoing was ‘no prospect of improvement’, 24% of physicians thought is was very important, 25% thought moderately important - The primary aim of foregoing was ‘avoid unnecessary prolonging of life’ (64%), and ‘optimising patient comfort’ (21%) - Most NHPs (70%), nurses (40%) and family members (46%) thought the family had considerable influence - Most NHPs (49%), nurses (55%) and family members (37%) thought the NHP had considerable influence | H H H - **H** |
| 19 | Pasman, H.R. *J Aging Stud,* 2004; 18(3): 321-335. | Netherlands | - Nursing homes (N.2) - Starting and withholding decisions - ANH non-specific - Dementia patients | Ethnography – participant observation within nursing homes | - Patients non-verbal expressions such as pushing food away were sometimes interpreted as wishes - Patients who stated they wanted to die were more frequently deemed incompetent than those who stated they wanted to live - Nurses feel responsible for food and water making their decisions about AN more complex - Most NHPs aimed for consensus with decisions, though felt they were ultimately responsible - Most NHPs not in favour of starting AN because it unnecessarily prolongs dementia | H H H - **H** |
| 20 | Rurup, M.L. *Patient Education and Counseling,* 2006; 61(3): 372-380. | Netherlands | - Physicians (N.107), nurses (N.148), relatives (N.136) - Starting and withholding decisions - ANH non-specific - Dementia patients | Questionnaire containing closed questions | - 73% of relatives, 51% of nurses and 5% of physicians fully agreed with the statement ‘an advance directive should always be followed’ - When asked if food refusal by the patient should be respected at all times, 15% of physicians fully agreed, 35% of nurses and 47% of relatives. - When asked if withholding ANH was neglectful, 80% of physicians and 85% of nurses fully disagreed. (Relatives not asked) | H H H - **H** |
| 21 | Shega, J.W. *Journal of Palliative Medicine*, 2003; 6(6): 885-893. | USA | - Physicians (N. 195) - Starting and withholding decisions - PEG feeding - Dementia patients | Postal questionnaire using patient vignettes | - The most important factor in the decision was the patients wishes (94%) - Other important factors were: family preference (94%), cognitive status (92%), prognosis (92%), physical function (90%) - 47% of physicians said nursing homes had asked for a PEG, 65% said nursing home concern influenced PEG - 74% disagreed that PEG should be the basic standard of care | H H H - **H** |
| 22 | Somogyi-Zalud, E. *Archives of Gerontology and Geriatrics,* 32 (2001): 101–111. | USA | - Surrogate decision-makers (N. 50) - Starting, withholding and withdrawing decisions - ANH non-specific - Dementia patients | Structured telephone questionnaire | - 8% of patients had advance directives - 8% of residents had clear knowledge of the patient’s wishes regarding AN - 25% of surrogates consented to AN despite the fact they thought the resident would be against it - 54% rated the resident’s quality of life as extremely poor - 78% thought AN was benefit to the resident - Of the 21 who gave a reason for choosing AN, 86% said prolonging life | H H H - **H** |
| 23 | The, A.M. *British Medical Journal,* 2002; 325(7376):1326. | Netherlands | - Patients (N. 35), physicians (N. 8), nurses (N. 43), and families (N. 32) - Withholding decisions - ANH non-specific - Dementia patients | Ethnography in two phases | - Slow deterioration considered natural course of dementia, rarely resulting in AN - Sudden or acute illness often resulting in AN - Living will, the patient’s verbal wishes and interpretation of the patient’s behaviour were all influential in decisions - Physicians had final say but took into account wishes of family | H H H - **H** |
| 24 | van Wigcheren, P.T. *Aging Clinical & Experimental Research,* 2007; 19(1): 26-34. | Netherlands | - Nursing home physicians (NHP) (N. 704) - Starting, withholding and withdrawing decisions - ANH non-specific - Dementia patients | Structured postal questionnaire | - The two most important considerations for starting AN were inadequate intake (44%) and swallowing issues (32%) - Most important overall considerations were: result of rehydration 49%, physical condition 50%, wishes of family 37%, (presumed) wishes of the patient 41% | H H M - **H** |
| 25 | Verelst, GSC. Tijdschr Gerontol Geriatr, 2006; 37(2): 51-58. | Netherlands | - Relatives of dementia patients (N.99) - Starting,/ foregoing decisions - ANH non-specific - Dementia patients | Structured questionnaires | - Relatives rated decision-making process as more satisfactory when they rated their own influence as higher - Relatives felt they received adequate information - Satisfaction did not change over time - Satisfaction was not altered by the patient’s death | MHH - H |
| 26 | Zanetti, O. *Journal of Geriatric Psychiatry,* 1996; 11(12): 1111-1116. | Italy | - Nurses (N. 178) - Starting decisions - NG feed - Dementia patients | Anonymous questionnaire with open-ended questions | - The majority of nurses (73%) were favourable to enteral feeding for demented patients who were unable to eat or refusing food - Factors considered in the decision were prolonging life, increasing quality of life, increasing patient autonomy and medical factors such as preventing aspiration | M H H - **H** |
| 27 | Asai, A. *Journal of Medical Ethics*, 1999; 25(4): 302-308. | Japan | - Physicians (N. 190) - Withholding, withdrawing decisions - ANH non-specific - ABI patients | Postal questionnaire using a patient case vignette | - When the patient had no advance directive and no family, physicians were less likely to withdraw ANH: 3% would withdraw if the patient required no other life sustaining treatment, 6% would if the patient developed respiratory failure - Physicians were most likely to withdraw when patient had AD requesting withdrawal and the family want to stop treatment: 17% would withdraw if the patient required no other life sustaining treatment, 21% would withdraw if the patient developed respiratory failure | H M M - **M** |
| 28 | Berger, J.T. *Archives of Internal Medicine*, 2011; 171(2): 178-179. | USA | - Surrogate decision-makers (N. 39) - Starting decisions - PEG feeding - Dementia patients | Questionnaire with closed questions | - 56% of decision-makers perceived PEG as in the patient’s best interests, 33% made a substituted judgment, 10% knew the patient’s wishes - 25% would consent to PEG even if it was against patient’s wishes. These surrogates were more likely to be older, a spouse, and Catholic - 38% would never consider withdrawing PEG, 58% would withdraw because of reduced quality of life, 3% would withdraw if there was no improvement in quality of life - 70% would want a PEG for themselves under similar circumstances | M M H – **M** |
| 29 | Craig, G.M. *Developmental Medicine and Child Neurology*, 2003; 45(3): 183-188. | UK | - Parents of children with disabilities (N. 22) - Starting decisions - PEG and NG feeding - ID and other patients | Semi-structured interviews | - Stigmatizing effect of the visibility of NG tubes, PEG seen as more discreet - Special significance of oral feeding between parent and child - Oral feeding as enjoyment and social time - Parents had a fear of complications from the surgery | H L L - **M** |
| 30 | Day, L*. Journal of Advanced Nursing*, 1995; 21(2): 295-298. | USA | - Nurses (N. 80) - Starting and withholding decisions - ANH non-specific - Dementia patients | Semi-structured interview using patient vignette | - More nurses chose to force artificial feeding than to withhold it: 55% cited beneficence, 50% cited reasons of the sanctity of life - Main ethical reasons not to feed were: patient autonomy (25%) and quality of life (25%) - For feeding were ANH would usually be held the reasons given were: patient’s family asks (40%), medical head orders (40%), all other staff would feed (40%) patient has stressed the sanctity of life (30%) | M M M – **M** |
| 31 | Demertzi, A. *Journal of Neurology*, 2011; Jun; 258(6): 1058-65 | * 32 European countries | - Healthcare professionals (N. 2,475) - Withdrawing decisions - ANH non-specific - ABI Patients | Closed-question questionnaire | - Two-thirds would withdraw AN from a patient in a chronic vegetative state, 82% wanted not to be kept alive if they imagined themselves in this condition - Less than a third would withdraw from a minimally conscious patient, though 70% would want it withdrawn from themselves in the same situation - Religious, older and female respondents were less likely to find the withdrawal of ANH in chronic VS acceptable | H L L - **M** |
| 32 | Dierickx, K. *Acta Neurochirurgica*, 1998; 140(5): 481-489. | Belgium | - Neurosurgeons, neurologists and rehabilitation doctors (N.208) - Withdrawing decisions - ANH non-specific - ABI Patients | Postal questionnaire | - 94% would withdraw if patient were to remain vegetative, 17% would if patient were severely disabled but able to communicate without speech, 6% would if patient were severely disabled but able to speak - Length of time the patient has been in a vegetative state and patient age were the most influential factors in the decision-making | M M M – **M** |
| 33 | Guerriere, D.N. *Developmental Medicine & Child Neurology*, 2003; 45(7): 470-476. | Canada | - Mothers of children with PEG tubes (N. 50) - Starting decisions - PEG feeding - ABI, ID and other patients | Semi-structured qualitative interview | - 32 mothers thought PEG might increase the suffering of their child - 30 mothers felt PEG might stigmatize their children - 29 mother’s felt consenting to PEG meant they had failed as mothers - 31 mothers felt their child’s weight and nutritional status would improve - 25 mothers reported feeling pressure from health professionals | H M L - **M** |
| 34 | Hanson, L.C. *Journal of Palliative Medicine*, 2008; 11(8): 1130-1134. | USA | - Physicians (N. 280) - Starting decisions - PEG feeding - ABI and other patients | Structured interviews | - 86% of physicians expected an improvement in nutrition from placing a PEG. 76% expected to prevent aspiration, 61% expected to prolong life - 38% of physicians expected the PEG to facilitate nursing home placement - ‘Who ultimately made the decision?’ 62% answered physician, 18% family, 11% patient, and 10% reported shared decision-making | H M M - **M** |
| 35 | Hasan, M. *Gerontology*, 1995; 41(6): 326-331. | UK | - Geriatricians (N. 45), speech therapists (N. 7), dieticians (N. 25), and nurses (N. 71) - Starting, withholding and conversion from NG to PEG decisions - PEG and NG feeding - Dementia and other patients | Postal questionnaire with closed questions | - 64% felt quality of life was the most important factor - The majority of all respondents in each professional category would refer for PEG due to both reversible and progressive dementia. The percentages ranged from 84-85% - 64% said the decision to refer PEG was multi-disciplinary, involving patients and carer | M M L - **M** |
| 36 | Healy, S. *Journal of Human Nutrition and Dietetics*, 2002; 15(6): 445-454. | Ireland | - Dieticians (N. 155) - Starting, withholding and withdrawing decisions - PEG feeding - Dementia and ABI patients | Postal questionnaire | - The main reasons for opposing PEG for dementia was the belief it would not improve quality of life (25%) and because the patient is refusing food (11%) - The most common reason to start PEG for dementia: it would be unethical, inhumane & neglectful not to - For PVS patients, 35% of dieticians said the family’s wishes were the most influential - For PVS, patients’ poor prognosis & remote possibility of improvement were the most common reasons for withdrawing (% not stated) | M H M - **M** |
| 37 | Hirakawa, Y. *nihon Ronen Igakkai Zasshi, 2004; 41(1): 99-104.* | Japan | - Elderly people over 65 years (N.123) - Dementia patients compared with non-demented - ANH non-specific - Starting decisions | Retrospective hospital records review | - ANH prevalent in both demented and non-demented groups - Significantly more dementia patients received ANH | MHL - M |
| 38 | Kruit, A. Ned Tijdschr Geneeskd, 1999; 143(27): 1401-1404 | Netherlands | - Nursing home patients (N.3) - Dementia and ABI patients - NG, PN and PEG - Decisions to start/forego | Case studies | - Family, nurse and patients’ previous wishes and current behaviour are incorporated into decision-making - Doctor makes final decision | LMH - M |
| 39 | Kwok, T*. Journal of Advanced Nursing*, 2007; 58(3): 256-262. | Hong Kong | - Family caregivers (N.51) - Withholding decisions - ANH non-specific - Dementia patients | Interviews in three stages: assessment of participants knowledge, questionnaires, vignettes | - 20% would forego in critical illness and 21 (41%) if the patient was in a coma. After education: 28% would forego in critical illness and 55% would forego for coma - Caregivers believed their own judgment was most important in making decisions (61% thought greatly important) - The next most important opinions were: the doctor (49% great importance) and patient’s own wishes (35% great importance) | H M M - **M** |
| 40 | Ladas, S.D. *Digestive Diseases*, 2002; 20(3-4):289-292 | USA | - Relatives of those with PEG (N. 55) - Starting decisions - PEG feeding - ABI and other patients | Telephone interview using a structured questionnaires | - 70.9% thought PEG would prolong the patient’s life, 56.4% thought it would positively affect the course of the patient’s underlying disease, 65.5% thought it would positively affect quality of life - Most decision-makers (87%) believed their decision was correct, but 3.6% said that the doctor incorrectly suggested PEG | M H M - **M** |
| 41 | Modi, S. *Omega: Journal of Death & Dying,* 2010; 62(1): 77-92. | USA | - Community members (N. 28) - Starting and withholding decisions - PEG feeding - Dementia patients | Focus groups | - All participants believed ‘they want to do the right thing’, this meant following the wishes of the patient - When patient’s wishes weren’t known more aggressive treatment was favoured - Some felt decisions should be made as a family - Some made reference to their own preferences - Food was an important part of both African-American and Caucasian communities - Feeding tubes were seen to prolong life | M M M - **M** |
| 42 | Sharp, H.M. *American journal of speech-language pathology*, 2009; 18(3): 222-230. | USA | - Speech-language pathologists (N. 326) - Starting decisions - PEG and NG feeding - Dementia patients | Postal questionnaire with closed questions and patient vignette | - More than 80% said influential factors were: patient’s wishes, risk of pneumonia, history of pneumonia and calorie intake - 85% would not recommend PEG if a patient had a living will expressing this as their preference - 74% said family preference was influential - 84.4% did not feel at risk of legal action - 65% perceived the primary care physician influenced their recommendation | H M M - **M** |
| 43 | Wilmot, S. *Nursing Ethics*, 2002; 9(6): 599-611. | UK | - Nursing and care staff (N. 12) - General decision-making - PEG feeding - Dementia patients | Focus groups with patient vignettes | - Distress caused by aspiration was portrayed as major decision factor - Nurses discussed the benefit of the PEG keeping the patient alive against the disadvantage of reduced quality of life - Also discussed the balance between the pleasure of eating and the risk of choking - Patient’s food refusal were seen by some autonomy and others saw it as a sign mental illness | M H M - **M** |
| 44 | Akerlund, B.M. *Omega: Journal of Death and Dying*, 1990; 21(1): 15-19. | Sweden | - Nursing staff (N. 5) - Starting decisions - PEG and NG feeding - Dementia patients | Semi-structured qualitative interview | - 4 interviewees though NG was most humane, 1 thought spoon-feeding - The caregivers felt PEG would be technically easier, less traumatic and provide nutrition - Caregivers felt spoon-feeding would provide opportunity for contact and love – but it was more difficult | M L L - **L** |
| 45 | Aparanji, K.P. *Journal of the American Medical Directors’ Association*, 2010; 11(6): 453-456. | USA | - Nursing home resident (N. 1) - Starting, withholding decisions - PEG feeding - Dementia patients | Case study | - Factors which prompted the decision-making process were: underweight, not eating, malnutrition, failure to thrive, dysphagia and aspiration - Patient refused procedure, this decision was questioned due to his capacity | L L L - **L** |
| 46 | Barratt, J. *Journal of Human Nutrition and Dietetics,* 2000; 13(1): 51-54. | UK | - Alzheimer’s patient (N. 1) - Starting decision - PEG feeding - Dementia patient | Case study | - Health and medical factors were considered in the decision-making process: weight loss, dysphagia, sore tongue - The clinicians also used evidence based decision-making and conducted further research into the issue - The clinicians also assessed the ethical issues surrounding tube feeding | L L M - **L** |
| 47 | Bito, S. *BMC Medical Ethics*, 2007; 19; 8:7. | Japan | - Physicians (N. 304) - Withdrawing, withholding decisions - PEG and NG feeding - Dementia and ABI patients | Internet based survey using patient vignettes | - When a family asks for PEG withdrawal 53% of physicians responded that they usually do not withdraw - 50% said they frequently consulted with other physicians, 42% said they do not | L L L - **L** |
| 48 | Brett, A.S*. Archives of Internal Medicine*, 2001; 161(5): 745-748. | USA | - Hospital inpatients (N.154) - Starting decisions - PEG and RIG feeding - Dementia and ABI patients | Retrospective hospital record review | - Surrogate decision-makers authorised the decision in 92.2% of cases but only one record documented a detailed discussion between physician and surrogate | M L L - **L** |
| 49 | Brotherton, A.M. *Clinical Nursing Research*, 2007; 16(4): 350-369. | UK | - Relatives of nursing home residents (N.8) - Starting, withholding, withdrawing decisions - PEG feeding - ABI patients | Semi-structured qualitative interview | - Participants discussed having little or no involvement, or choice in the decision-making process for PEG insertion - Participants were dependent upon the medical team to make decisions | L L M - **L** |
| 50 | Callahan, C.M. *Journal of the American Geriatrics Society*, 1999; 47(9):1 105-1109. | USA | - Hospital patients or their surrogate decision-makers (N.55) - Starting decisions - PEG feeding - Dementia, ABI and other patients | Semi-structured interviews | - 56% reported staff actively involved the informant in the decision-making - 51% reported discussing their decision with others - Decision-making process initiated by medical personnel in conjunction with an acute medical event in 73% of cases - Initiated by extended care facility personnel due to not eating in 16% - Only in one case was the issue first raised by the family | M L L - **L** |
| 51 | Cogen, R. *Archives of Internal Medicine*, 152: 1885-1888. | USA | - Family members of nursing home residents (N.102) - Starting, withholding decisions - ANH non-specific - Dementia patients | Postal questionnaire | - 3.1% said they took previous statements made by the patient (either written or oral) into account when making decisions - 5.1% said they assumed what the resident would want, 30.6% said they used their own personal views, 61.2% said they used a combination | M L L - **L** |
| 52 | Detweiler, M.B. *American Journal of Alzheimer's Disease and Other Dementias*, 2004; 19(1): 24-30. | USA | - Dementia patients (N. 3) - Starting and withholding decisions - PEG feeding - Dementia and ABI patients | Case studies | - Health factors such as aspiration were considered in all cases - Patient’s behaviour such as pulling out the gastrostomy tube was taken into account in two cases - Family wishes were taken into account in two cases | L L M - **L** |
| 53 | Enrione, E.B. *Journal of the American Dietetic Association*, 2007; 107(3): 416-421. | USA | - Registered dieticians (N. 499), and nurses (N. 471) - Starting decisions - ANH non-specific - Dementia patients | Postal questionnaire using patient vignettes and belief statements | - A younger age (62 vs. 82 years) and a ‘happy’ were most likely to be fed by both professional groups - Registered dieticians made the decision to feed more frequently than the nurses | M L L - **L** |
| 54 | Hicks, M.H. *Culture, Medicine and Psychiatry*, 23(4): 415-452. | USA | - Chinese-American families (N. 7) - General decision-making - ANH non-specific - Dementia and ABI patients | Semi-structured qualitative interview | - Caregivers named four major types of decision-makers: family members, health providers, social service staff or agencies and the ill elders - 5 of the 7 families made decisions as a family - Families struggled over power in decisions – gender was sometimes an issue | M L L - **L** |
| 55 | Hollows K. *Nursing Praxis New Zealand*, 1995; 10(1):28-37. | New Zealand | - Nurses (N. 5) - Withdrawing decisions - PEG feeding - ABI patients | Qualitative interview | - Most decisions involved a multi-disciplinary team, in one case the decision went to the ethics committee - In-house decision-making was preferred | L L L - **L** |
| 56 | Kowalski, S. *Gastroenterology nursing: the official journal of the Society of Gastroenterology Nurses and Associates,* 1996; 19(1): 25-28. | USA | - PVS patients (N. 1) - Withdrawing decisions - PEG feeding - ABI patients | Case study | - The patient’s spoken wishes were taken into account - The family considered the ethics of letting the patient ‘starve to death’ - Family felt a PEG would not add to the patient’s quality of life | L L M - **L** |
| 57 | Kwok, T*. International Journal of Geriatric Psychiatry*, 2001; 16(3): 337-338. | Hong Kong | - Dementia patient (N. 1) - Starting, withholding decisions - PEG feedings - Dementia patients | Case study | - Food refusal and loss of appetite were decision triggers - The distress and discomfort of the NG tube was considered | L L L - **L** |
| 58 | Lacey, D*. American Journal of Alzheimer's Disease & Other Dementias*, 2005; 20(4): 211-220. | USA | - Nursing home social services staff (N. 138) - Starting and withholding decisions - PEG feeding - Dementia patients | Questionnaire | - 45% of staff thought the medical director had the most influence - 25% thought the director of nursing had the most influence | L L L - **L** |
| 59 | Lee, L. *Internal Medicine Journal,* 2010; 40(6): 411-418. | Australia | - Young people with profound multiple disabilities (N. 40) - Starting and continuing decisions - PEG feeding - Intellectual disability (ID) patients | Retrospective record audit and observational study | - PEG feeding was initiated due to as varying combinations of gastro-oesophageal reflux (20 people) and aspiration/ coughing (30 people) - Other decision factors were prolonged feeding times (14 people) and loss of weight in the previous 12 months (20 people) - Only one family member felt fully informed and involved in the decision. Most felt decision had already been made and that they had no choice | M L L - **L** |
| 60 | Leicher, C.R. *Archives of Pediatric Adolescent Medicine*, 1994; 148(1):87-92. | USA | - Child with PVS (N. 1) - Withdrawing decision - PEG feeding - ABI patient | Case study | - The child’s parents felt she had no quality of life - The parents’ experience with PVS influenced their decision | L L L - **L** |
| 61 | Scott, L.D. *American Journal of Gastroenterology,* 2005; 100(4): 740-743. | USA | - Elderly male (N. 1) - Starting, withholding decision - PEG feeding - Dementia and ABI patients | Case study | - The patient’s inability to eat or drink triggered the decision-making - Professional relationships and expectations were considered by the physician in the decision-making - The patient’s informal wishes not receive a PEG were taken into account | L L L - **L** |
| 62 | Shah, S.H. *Palliative Medicine*, 2006; 20(7): 711-714. | UK | - Alzheimer’s patient (N. 1) - Starting, withholding decisions - PEG feeding - Dementia patients | Case study | - Inadequate oral intake and suspicion of dysphagia triggered the decision-making process - Poor prognosis and no expected benefits were the key decision-making factors | L L L - **L** |
| 63 | Simionato, L. *Journal of Hospice & Palliative Nursing*, 2010; 12(6): 378-385. | Italy | - Physicians (N. 35) and nurses (N. 80) - General decision-making - PEG and NG feeding - Dementia patients | Questionnaire using closed questions | - Most decisions were made by the team (43% reported for doctors and 54% for nurses) or only by one team member (30.6% for doctors and 54% for nurses) - 11.4% of the decisions were made by the family alone, and 10% by family and team | M L L - **L** |
| 64 | Solarino, B. *Intensive Care Medicine*, 2011; Mar;37(3): 542-9. | Italy | - Physicians (N. 22,219) - Withholding, withdrawing decisions - ANH non-specific - ABI patients | Email questionnaire using closed questions | - 67% of respondents believed that a patient should have the right to refuse ANH - 35% of participants were unfamiliar with the concept of advance directives | M L L - **L** |
| 65 | Tresch, D.D. *Journal of the American Geriatric Society*, 1991 Jan; 39(1): 17-21. | USA | - Family of patients with PEG tubes (N. 33) - Starting, withdrawing and continuing decisions - PEG feeding - Dementia and ABI patients | Questionnaire with closed questions | - 25 families felt PEG should not be removed unless another method of feeding could be provided - 29 of the 33 family members retrospectively agreed with the decision to start the tube feeding | M L L - **L** |
| 66 | Wall, M.G. *Journal of the American Dietetic Association*, 1991; 91(5): 549-552. | USA | - Dieticians (N. 250) - Withdrawing and continuing decisions - PEG, NG and TPN feeding - ABI and other patients | Postal questionnaire | - TPN was most likely to be discontinued when death was imminent - Dieticians were more likely to agree to stop feeding because of a patient’s request than because of a guardian’s - Dieticians disagreed cost should be a factor - The most agreed with factor was that feeding should be stopped if it causes the patient pain | M L L - **L** |

* Austria, Belgium, Bulgaria, Croatia, Cyprus, Czech Republic, Denmark, Estonia, Finland, France, Germany, Greece, Hungary, Italy, Lithuania, Luxembourg, Moldavia, Netherlands, Norway, Poland, Portugal, Romania, Russia, Serbia, Slovakia, Slovenia, Spain, Sweden, Switzerland, Turkey, United Kingdom, the former Yugoslav Republic of Macedonia.
