# Supplementary material for: How are Treatment Decisions Made about Artificial Nutrition for Individuals at Risk of Lacking Capacity? A Systematic Literature Review
Source: PLoS One. 2013 Apr 16;8(4):e61475. doi: 10.1371/journal.pone.0061475 (PMC3628879; doi:10.1371/journal.pone.0061475)
Supplement: File S2 — Flowchart illustrating the search process (DOCX) [file pone.0061475.s003.docx]

**File S2. Flowchart illustrating the search process**
