# Supplementary material for: How are Treatment Decisions Made about Artificial Nutrition for Individuals at Risk of Lacking Capacity? A Systematic Literature Review
Source: PLoS One. 2013 Apr 16;8(4):e61475. doi: 10.1371/journal.pone.0061475 (PMC3628879; doi:10.1371/journal.pone.0061475)
Supplement: Materials S1 — Example of one search strategy (DOCX) [file pone.0061475.s004.docx]

**Materials S1. Example of one electronic search strategy**

Electronic search strategies were developed in with a librarian (IK). Illustrated below is the search string from one database PubMed. Which produces 3433 results.

((("Enteral Nutrition"[Mesh]) OR ("Parenteral Nutrition"[Mesh]) OR ("Gastrostomy"[Mesh]) OR ("Feeding methods"[MeSH]) OR ("Nutritional support"[MeSH]) OR (artificial nutrition[Title/Abstract]) OR (Artificial feeding[Title/Abstract]) OR (Artificially fed[Title/Abstract]) OR (PEG[Title/Abstract]) OR (percutaneous endoscopic gastrostom*[Title/Abstract]) OR (ANH[Title/Abstract]) OR (Feeding methods[Title/Abstract]) OR (Nutritional support[Title/Abstract]) OR (stamm gastrostom*[Title/Abstract]) OR (Enteral Nutrition[Title/Abstract]) OR (Naso gastric tube[Title/Abstract]) OR (NG tube[Title/Abstract]) OR (Naso gastric feed*[Title/Abstract]) OR (Total parenteral nutrition[Title/Abstract]) OR (TPN[Title/Abstract]) OR (parenteral nutrition[Title/Abstract])) AND (("Decision Making"[Mesh]) OR ("Supreme Court Decisions"[Mesh]) OR ("Resuscitation Orders"[Mesh]) OR ("Withholding treatment"[MeSH]) OR ("Advance directives"[MeSH]) OR (Withholding treatment[Title/Abstract]) OR (Decision making[Title/Abstract]) OR (Decision*[Title/Abstract]) OR (Choice[Title/Abstract]))) OR ((("Mental Retardation"[Mesh]) OR ("Learning Disorders"[Mesh]) OR (Intellectual* AND disab*[Title/Abstract]) OR (Learning disab*[Title/Abstract])) AND (("Enteral Nutrition"[Mesh]) OR ("Parenteral Nutrition"[Mesh]) OR ("Gastrostomy"[Mesh]) OR ("Feeding methods"[MeSH]) OR ("Nutritional support"[MeSH]) OR (artificial nutrition[Title/Abstract]) OR (Artificial feeding[Title/Abstract]) OR (Artificially fed[Title/Abstract]) OR (PEG[Title/Abstract]) OR (percutaneous endoscopic gastrostom*[Title/Abstract]) OR (ANH[Title/Abstract]) OR (Feeding methods[Title/Abstract]) OR (Nutritional support[Title/Abstract]) OR (stamm gastrostom*[Title/Abstract]) OR (Enteral Nutrition[Title/Abstract]) OR (Naso gastric tube[Title/Abstract]) OR (NG tube[Title/Abstract]) OR (Naso gastric feed*[Title/Abstract]) OR (Total parenteral nutrition[Title/Abstract]) OR (TPN[Title/Abstract]) OR (parenteral nutrition[Title/Abstract]))) OR ((("Dementia"[Mesh]) OR (Dementia[Title/Abstract]) OR (Alzheimer*[Title/Abstract]) OR (Cognitive impair*[Title/Abstract])) AND (("Enteral Nutrition"[Mesh]) OR ("Parenteral Nutrition"[Mesh]) OR ("Gastrostomy"[Mesh]) OR ("Feeding methods"[MeSH]) OR ("Nutritional support"[MeSH]) OR (artificial nutrition[Title/Abstract]) OR (Artificial feeding[Title/Abstract]) OR (Artificially fed[Title/Abstract]) OR (PEG[Title/Abstract]) OR (percutaneous endoscopic gastrostom*[Title/Abstract]) OR (ANH[Title/Abstract]) OR (Feeding methods[Title/Abstract]) OR (Nutritional support[Title/Abstract]) OR (stamm gastrostom*[Title/Abstract]) OR (Enteral Nutrition[Title/Abstract]) OR (Naso gastric tube[Title/Abstract]) OR (NG tube[Title/Abstract]) OR (Naso gastric feed*[Title/Abstract]) OR (Total parenteral nutrition[Title/Abstract]) OR (TPN[Title/Abstract]) OR (parenteral nutrition[Title/Abstract]))) OR ((("Brain Injuries"[Mesh]) OR (Head injur*[Title/Abstract]) OR (Brain injur*[Title/Abstract]) OR (ABI[Title/Abstract]) OR (vegetative state[Title/Abstract]) OR (PVS[Title/Abstract])) AND (("Enteral Nutrition"[Mesh]) OR ("Parenteral Nutrition"[Mesh]) OR ("Gastrostomy"[Mesh]) OR ("Feeding methods"[MeSH]) OR ("Nutritional support"[MeSH]) OR (artificial nutrition[Title/Abstract]) OR (Artificial feeding[Title/Abstract]) OR (Artificially fed[Title/Abstract]) OR (PEG[Title/Abstract]) OR (percutaneous endoscopic gastrostom*[Title/Abstract]) OR (ANH[Title/Abstract]) OR (Feeding methods[Title/Abstract]) OR (Nutritional support[Title/Abstract]) OR (stamm gastrostom*[Title/Abstract]) OR (Enteral Nutrition[Title/Abstract]) OR (Naso gastric tube[Title/Abstract]) OR (NG tube[Title/Abstract]) OR (Naso gastric feed*[Title/Abstract]) OR (Total parenteral nutrition[Title/Abstract]) OR (TPN[Title/Abstract]) OR (parenteral nutrition[Title/Abstract]))) AND (("1990/01/01"[PDat] : "2011/11/30"[PDat]))
