# Supplementary material for: How are Treatment Decisions Made about Artificial Nutrition for Individuals at Risk of Lacking Capacity? A Systematic Literature Review
Source: PLoS One. 2013 Apr 16;8(4):e61475. doi: 10.1371/journal.pone.0061475 (PMC3628879; doi:10.1371/journal.pone.0061475)
Supplement: Materials S2 — Research protocol (DOCX) [file pone.0061475.s005.docx]

**Materials S2. Research protocol**

**How are Treatment Decisions made about Artificial Nutrition for Individuals at Risk of Lacking Capacity? A Systematic Literature Review (Protocol)**

**Background**

Decision-making capacity is a psychological construct which refers to a person’s ability to understand and balance the necessary information, and to communicate a choice. It has been enshrined in law in some countries, such as England and Wales (Mental Capacity Act 2005). Across the world, the number of people at risk of lacking the capacity to participate in healthcare decisions is rising. Decision-making for these individuals is challenging. It is particularly when questions of capacity arise with respect to the maintenance of life, or the end of life, as exemplified by decisions concerning artificial means for maintaining nutrition, that making choices may be the most difficult and contentious. As this population continues to grow, making treatment decisions for these individuals is becoming an increasingly important issue for clinical practice and policy worldwide.

**Aims**

Using the exemplar of decisions regarding artificial nutrition, we will undertake a systematic review and qualitative synthesis of the international evidence on treatment decisions for those at risk of lacking capacity. We will compare and contrast the different issues for three groups of patients who represent similar and different issues surrounding capacity; those with dementia, intellectual disabilities and acquired brain injuries. The aim is to help inform decision-making in clinical practice and healthcare policy worldwide.

**Objectives**

To undertake a systematic literature review and qualitative synthesis of treatment decisions concerning artificial nutrition for individuals at risk of lacking capacity due to dementia, intellectual disability, or acquired brain injury. With regard to:

a) How decisions were made

b) Who was involved in the decision-making

c) Which factors were considered

**Methods**

**Criteria for selection of studies: Inclusion criteria**

**Types of studies:** Studies are to be included only if they involve original empirical evidence. All types of study design will be incorporated, including both quantitative and qualitative designs. Studies involving a medical case study design will also be incorporated. Studies about any method of artificial nutrition are to be included.

Studies will only be included if they involve treatment decision-making for individuals who lack capacity, or risk lacking capacity, from dementia, intellectual disability or acquired brain injury. Studies will also be included if the individuals have more than one condition, as long one of their diagnoses is either dementia, intellectual disability or acquired brain injury.

**Types of participants:** Studies involving any type of participant will be included, as long as the participant has been involved in at least one aspect of decision-making. The participants could be physicians, nurses, family members, the patient themselves, or any other individual. There are no restriction on age, studies can be included if they involved children or adults.

**Types of outcomes:** Studies will be included as long they collect and report data about any aspect of the decision-making process, this could involve decisions to start, withdraw, forego and/or replace artificial nutrition. There are no restrictions set on the types of measures used for outcomes, all measures can be incorporated.

**Research time frame:** Studies are to be included if they were published over the twenty year period between January 1990 and November 2011.

**Exclusion criteria**

Studies about preferences for artificial nutrition should be excluded if they do not contain data about decision-making or the decision-making process. Summary papers, opinion and commentary pieces, newspaper articles and reviews are to be excluded. Legal case studies are excluded.

**Electronic searches and information sources**

Searching for qualitative literature is notoriously difficult, we will use search criteria for both qualitative and quantitative studies developed in collaboration with a librarian, or information specialist. The six electronic databases to be searched are:

1. PubMed
2. AMED
3. CINAHL
4. EMBASE
5. PsychINFO
6. OpenSigle

**Other searches**

Hand searches of key journals identified through the process of searching will be undertaken, and citation searches of selected articles will also be undertaken.

**Quality evaluation and sensitivity analysis**

To assess paper quality, relevance, and contribution, Gough’s Weight of Evidence framework will be utilised as it provides a useful flexible framework which can be applied to many research designs. Two to three researchers will independently weight the studies. All weights of study will be included for the final analysis provided they meet the minimal requirements for relevance and quality. A sensitivity analysis will be performed to examine the effect of removing studies rated ‘high’ or ‘low’ on the research findings.

**Data extraction**

Data will be extracted using a standardised data extraction form by two researchers independently.

**Data analysis and synthesis**

To analyse and synthesis the data, Thomas and Harden’s (2008) ‘thematic synthesis’ will be utilised to develop descriptive themes into analytical themes in the NVivo qualitative analysis software. Analytical themes will be developed in an iterative process of discussion, analysis and consultation among the researchers.
